# Short-term changes of intraocular pressure and ocular perfusion pressure after intravitreal injection of bevacizumab or ranibizumab

**DOI:** 10.1186/s12886-016-0255-8

**Published:** 2016-05-31

**Authors:** Jong Wook Lee, Hoon Park, Jeong Han Choi, Hyun Joo Lee, Sang Woong Moon, Ja Heon Kang, Young Gyun Kim

**Affiliations:** Department of Ophthalmology, School of Medicine, Eulji University, Daejeon, Korea; Department of Ophthalmology, Kyung Hee University Hospital at Gangdong, School of Medicine, Kyung Hee University, #892 Dongnam-ro, Gangdong-gu, Seoul Korea

**Keywords:** Intraocular pressure, Mean ocular perfusion pressure, Intravitreal injection

## Abstract

**Background:**

The aim of this study was to investigate the effect of intravitreal anti-vascular endothothelial growth factor (VEGF) injection on intraocular pressure (IOP) and mean ocular perfusion pressure (MOPP).

**Methods:**

MOPP results were obtained by measuring mean arterial pressure (MAP) and IOP just before the injection, immediately after the injection, at 30 min, 1 day, and 1 week after injection from 65 eyes of 42 patients.

**Results:**

Pre-injection mean IOP was 16.66 ± 3.50 mmHg, and mean IOP was 43.81 ± 9.69 mmHg immediately after the injection, 17.57 ± 4.44 mmHg at 30 min, 15.00 ± 4.21 mmHg at 1 day, and 15.90 ± 3.63 mmHg at 1 week after the injection. Pre-injection mean MOPP was 46.39 ± 5.78 mmHg, and mean MOPP was 25.14 ± 8.79 mmHg immediately after the injection, 45.87 ± 6.31 mmHg at 30 min, 46.93 ± 6.25 mmHg at 1 day, and 46.50 ± 4.94 mmHg at 1 week after the injection.

**Conclusion:**

The instant increase in IOP by intravitreal anti-VEGF injection led to a transient decrease in MOPP. Based on this finding, the instant increase in IOP after intravitreal anti-VEGF injection does not significantly impair retinal blood flow.

## Background

Intravitreal injection is an excellent method for delivering drugs for various vitreoretinal diseases, the incidences of which are increasing globally every year. Various vitreoretinal diseases such as choroidal neovascularization due to exudative age-related macular degeneration, diabetic macular edema, and macular edema due to retinal vein occlusion are treated with anti-vascular endothelial growth factors (VEGF) such as ranibizumab (Lucentis®) and bevacizumab (Avastin®). Thus, expansion of the use of intravitreal anti-VEGF is expected.

The volume of the intravitreal injection can directly increase the intraocular pressure (IOP) and thereby negatively affect the retinal and optic nerve blood supply. Animal experiments show that an acute rise in IOP blocks axonal transport to the optic nerve head, and damage to the optic nerve is directly proportional to the rise in IOP [1]. An acute rise in IOP also decreases juxtapapillary retinal and optic nerve head blood flow proportional to the quantitative rise in IOP [2].

There are many reports about safe levels of an increase in IOP after intravitreal anti-VEGF injection [3–7]. Studies of Lucentis® (ranibizumab, Genentech Inc./Roche, South San Francisco, CA. USA) revealed that there was no long-term change in IOP over 2 years of follow-up [8]. IOP increased during the first hour post-injection and then dropped within 60 min to only 2 to 3 mmHg higher than the baseline level. This study also reported that at 1 h post-injection, 18 % of patients had an IOP of 30 mmHg or higher. The report on escalating doses of Lucentis showed no changes in IOP for up to 4 months of follow-up [9].

Avastin® (bevacizumab, Novartis/Genentech Inc., CA. USA) is a drug used off-label for intravitreal treatment of various retinal diseases. A previous report on the levels of post-injection IOP demonstrated that intravitreal Avastin causes a volume-related increase in IOP that does not occlude the central retinal artery. The IOP fell below 30 mmHg in all selected eyes within 15 min [5].

Although previous studies focused on the rise in post-injection IOP, substantially the entire blood supply of the retina and optic nerve head is affected by ocular perfusion pressure, which means that ocular perfusion pressure is more important than IOP. Mean ocular perfusion pressure (MOPP) is defined as the difference between the mean ophthalmic artery pressure and IOP [10]. Clinical measurement of the mean pressure of the ophthalmic artery, however, is quite difficult. The mean ophthalmic artery pressure can be estimated by using a value of two-thirds of the mean blood pressure (MBP) of the brachial artery measured in a seated position [11]. Therefore, in the present study, we defined the MOPP as the difference between 2/3*MBP of the brachial artery and IOP [10]. The normal range of MOPP is 40 mmHg to 55 mmHg.

A volume-related IOP increase due to intravitreal anti-VEGF injection will decrease the MOPP and negatively affect the retina and optic nerve head blood supply. The purpose of this study was to analyze the short-term change in IOP and MOPP after intravitreal anti-VEGF injection, and to evaluate the safety of the procedure.

## Methods

This study was a prospective case series of consecutive patients undergoing intravitreal injection of anti-VEGF at the Eulji Medical Center, Seoul, Korea from October 2011 to November 2011. Patients were asked to participate if they were over 20 years of age and were able to have a regular follow-up. To maximize generalizability, exclusion criteria were kept to a minimum. Patients who had intravitreal injections of triamcinolone acetonide any time up until 6 months prior to the study, patients with active uveitis or ocular ischemic syndrome, and diagnosed with any type of glaucoma were excluded from selection. According to these exclusion criteria, 103 eyes of 67 patients were excluded from a total 168 eyes of 109 patients. But the patients participating in this study would have undergone anti-VEGF intravitreal injection even if they had not been enrolled.

The prospective clinical study was approved by the Institutional Review Board (IRB)/Ethics Committee at the Eulji Medical Center of Eulji University, Seoul, Korea. Informed consent was obtained from each patient prior to participation in the study. All procedures conformed to HIPAA regulations and the Declaration of Helsinki for research involving human subjects.

Before intravitreal injection, patients signed a standard informed consent form describing the potential risks and benefits of the procedure and consecutive management, which included IOP and blood pressure measurements.

The following basic tests were made on every subject before going to operation room for intravitreal injection: (1) best corrected visual acuity and IOP, (2) blood pressure, (3) slit-lamp examination. Patients who had one of the following conditions underwent the delayed procedure: (1) systolic blood pressure (SBP) over 140 mmHg, (2) diastolic blood pressure (DBP) over 90 mmHg, and (3) IOP over 30 mmHg.

Intravitreal injections were performed by one surgeon (Young Gyun Kim) using aseptic techniques in an operating room at an outpatient ophthalmology clinic. Patients were prepared and draped in a standard fashion and positioned supine for the procedure. After applying topical proparacaine for anesthesia, a lid speculum was used for lid control, and the eyeball was sterilized with a solution of 5 % povidone–iodine and irrigated with sterile BSS. Intravitreal injection of Avastin (bevacizumab, 0.05 mL) or Lucentis (ranibizumab, 0.07 mL) was injected with a 30-guage needle through the inferotemporal or inferonasal pars plana 3.5 mm posterior to the limbus. The needle was inserted approximately 1.0 cm into the globe, and the injection was performed. After injection, a sterile cotton swab was placed on the injection site to prevent reflux of the medicine or vitreous.

MBP and IOP values were obtained at baseline, immediately after the injection, and at 30 min, 1 day, and 1 week after injection. The MBP of the brachial artery was measured with an automatic sphygmomanometer with the patient in the sitting position, and the IOP was measured in the same position with an Icare® PRO rebound tonometer model (Icare, Tiolat Oy, Helsinki, Finland) by the same examiner. MOPP was measured using the following formula [12]. MOPP = 2/3 * [diastolic pressure + 1/3*(systolic pressure—diastolic pressure)]—IOP. Thus, MOPP was calculated using the measured BP and IOP at each time-point.

Statistical tests were performed using SPSS software version 14.0 (SPSS Inc, Chicago, IL) The baseline values and individual values of IOP and MOPP at each time-point were analyzed using a paired *t*-test. Variable factors that can affect IOP and MOPP after intravitreal injection—the difference in dosage (Avastin 0.05 mL, Lucentis 0.07 mL) and the presence of post-injection vitreous regurgitation—were set and each group was analyzed using the Student’s *t*-test.

## Results

The data for 65 eyes of 42 patients were included in this analysis. Table 1 shows the basic patient characteristics and clinical data for the sample. Mean patient age was 59.7 ± 13.6 years. Classifying the patients by disease type resulted in: 16 eyes (24.6 %) with diabetic macular edema, 10 eyes (15.4 %) with proliferative diabetic retinopathy-vitreous hemorrhage (a large majority had diabetes mellitus (DM)-related disease), 15 eyes (23.1 %) with exudative age-related macular degeneration, 14 eyes (21.5 %) with retinal vein occlusion-related macular edema, 8 eyes (12.3 %) with chronic central serous chorioretinopathy, and 2 eyes (3.1 %) with idiopathic choroidal neovascularization. Intravitreal injection of Avastin (0.05 mL) or Lucentis (0.07 mL) was performed in 50 eyes (76.9 %) or 15 eyes (23.1 %), respectively. Post-injection vitreous regurgitation was confirmed in 11 (16.9 %) of 65 eyes.

**Table 1:** Demographic and Clinical Characteristics of the Study Sample

Variable	No. (%)
Sex (No. of patient)	
Female	18 (42.9 %)
Male	24 (57.1 %)
Age, years (No. of patients)	
20–59	21 (50.0 %)
60–69	10 (23.8 %)
70–79	9 (21.4 %)
80+	2 (4.8 %)
Diagnosis (No. of eyes)	
DME	16 (24.6 %)
PDR—vitreous hemorrhage	10 (15.4 %)
AMD/Wet type	15 (23.1 %)
RVO related ME	14 (21.5 %)
Chronic CSC	8 (12.3 %)
Idiopathic CNV	2 (3.1 %)
Drug (No. of eyes)	
Bevacizumab (0.05 mL)	50 (76.9 %)
Ranibizumab (0.07 mL)	15 (23.1 %)
Postoperative vitreous regurgitation (No. of eyes)	
Yes	11 (16.9 %)
No	54 (83.1 %)

*DME* diabetic macular edema, *PDR* proliferative diabetic retinopathy, *AMD* age-related macular degeneration, *RVO* retinal vein occlusion, *CSC* central serous chorioretinopathy, *CNV* choroidal neovascularization

**Fig. 1:**
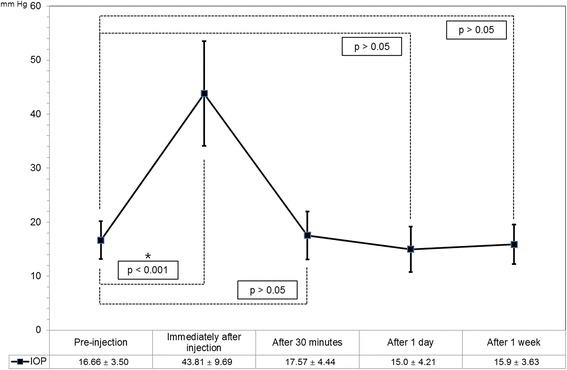
Short-term changes in intraocular pressure (IOP) after intravitreal Avastin or Lucentis injection. Compared to the pre-injection IOP values, only those checked immediately after injection were significantly different

**Fig. 2:**
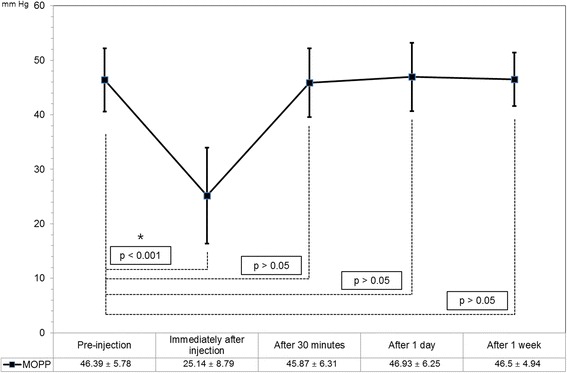
Short-term changes in mean ocular perfusion pressure (MOPP) after intravitreal Avastin or Lucentis injection. Compared to the pre-injection MOPP values, only those checked immediately after injection were significantly different

Figure 1 shows the change in IOP beginning from the value before the injection to the value of 4 post-injection time points. Pre-injection mean IOP was 16.66 ± 3.50 mmHg, and it was 43.81 ± 9.69 mmHg immediately after the injection, 17.57 ± 4.44 mmHg at 30 min, 15.00 ± 4.21 mmHg at 1 day, and 15.90 ± 3.63 mmHg at 1 week after the injection. Figure 2 shows the change in MOPP beginning from the value before the injection to the value of 4 post-injection time-points. Pre-injection mean MOPP was 46.39 ± 5.78 mmHg, and it was 25.14 ± 8.79 mmHg immediately after the injection, 45.87 ± 6.31 mmHg at 30 min, 46.93 ± 6.25 mmHg at 1 day, and 46.50 ± 4.94 mmHg at 1 week after the injection.

Only the IOP immediately after injection was significantly different from the pre-injection IOP (Fig. 1, *p* < 0.001). Only the MOPP immediately after the injection was significantly different from the pre-injection MOPP value (Fig. 2, *p* < 0.001).

The IOP and MOPP recovered to their pre-injection values within 30 min after injection. 98.5 % of the IOP values recovered to less than 30 mmHg within 30 min after injection (Table 2) and 87.7 % of MOPP values recovered to above 40 mmHg within 30 min after injection (Table 3).

**Table 2:** Intraocular Pressure (IOP) at Baseline and at Various Times after Intravitreal Injection

IOP range (mmHg)	No. (%)				
	Baseline	Immediately	30 min	24 h	1 week
0–9	1 (1.5 %)		1 (1.5 %)	2 (3.1 %)	
10–19	54 (83.1 %)		42 (64.6 %)	59 (90.8 %)	59 (90.8 %)
20–29	10 (15.4 %)	5 (7.7 %)	21 (32.3 %)	4 (6.1 %)	6 (9.2 %)
30–39		15 (23.1 %)	1 (1.5 %)		
40–49		22 (33.8 %)			
50–59		20 (30.8 %)			
60–69		3 (4.6 %)

**Table 3:** Mean Ocular Perfusion Pressure (MOPP) at Baseline and at Various Times after Intravitreal Injection

MOPP range (mmHg)	No. (%)				
	Baseline	Immediately	30 min	24 h	1 week
0–9					
10–19		14 (21.6 %)			
20–29		30 (46.2 %)	1 (1.5 %)	2 (3.1 %)	5 (7.7 %)
30–39	9 (13.8 %)	19 (29.2 %)	7 (10.8 %)	1 (1.5 %)	43 (66.1 %)
40–49	36 (55.4 %)	1 (1.5 %)	35 (53.8 %)	39 (60.0 %)	15 (23.1 %)
50–59	18 (27.7 %)	1 (1.5 %)	22 (33.8 %)	22 (33.8 %)	2 (3.1 %)
60–69	2 (3.1 %)			1 (1.5 %)	

In addition, the patients were divided into two groups according to the injection type (0.05 mL Avastin or 0.07 mL Lucentis) and their IOP and MOPP values immediately after injection were analyzed. We also evaluated the presence of post-injection vitreous regurgitation. The mean IOP of the Avastin group was 41.72 ± 9.74 mmHg and that of the Lucentis group was 45.15 ± 9.65 mmHg. The mean MOPP of the Avastin group was 26.81 ± 8.68 mmHg and that of the Lucentis group was 24.27 ± 8.51 mmHg. IOP and MOPP values were not significantly different between the Avastin group and Lucentis group (Table 4, *p* > 0.05).

**Table 4:** Intraocular Pressure (IOP) and Mean Ocular Perfusion Pressure (MOPP) immediately after the Injection depending on the Type of Injection

Variable	Average IOP immediately after the Injection (mmHg)	Average MOPP immediately after the Injection (mmHg)
Drug		
Bevacizumab (0.05 mL)	41.72 ± 9.74	26.81 ± 8.68
Ranibizumab (0.07 mL)	45.15 ± 9.65	24.27 ± 8.51
*p* value	> 0.05	> 0.05

Mean IOP was 39.92 ± 9.12 mmHg in the group with post-injection vitreous regurgitation and 44.68 ± 9.27 mmHg in the group without post-injection vitreous regurgitation. Mean MOPP was 28.83 ± 9.56 mmHg in the group with post-injection vitreous regurgitation and 24.48 ± 8.27 mmHg in the group without post-injection vitreous regurgitation. Post-injection vitreous regurgitation did not significantly change mean MOPP and IOP (Table 5, *p* > 0.05).

**Table 5:** Intraocular Pressure (IOP) and Mean Ocular Perfusion Pressure (MOPP) immediately after the Injection depending on the Post-injection Vitreous Regurgitation

Variable	Average IOP immediately after the Injection (mmHg)	Average MOPP immediately after the Injection (mmHg)
Post-injection vitreous regurgitation		
Yes	39.92 ± 9.12	28.83 ± 9.56
No	44.68 ± 9.27	24.48 ± 8.27
*p* value	> 0.05	> 0.05

## Discussion

As intravitreal anti-VEGF injection becomes a more common treatment modality for various vitreoretinal diseases, there is increased concern regarding acute IOP elevation after intravitreal injection in relation to the acute increase in volume inside the eye. Several studies have examined post-injection IOP spikes and most revealed that IOP spikes are transient and additional intervention to lower the IOP, such as anterior chamber paracentesis, is not necessary [4–9]. Previous studies focused only on the rise in post-injection IOP. The blood supply to the retina and optic nerve head, however, is affected more by a change in the ocular perfusion pressure than by an increase in IOP. The present study is one of the first studies to examine the decrease in MOPP that occurs following intravitreal injection in addition to the post-injection increase in IOP.

The blood supply to the retina and optic nerve head is maintained by autoregulation, which means proper microcirculation in these tissues is predicted to be spared despite a sudden decrease in MOPP. There are few studies of human eyes regarding this issue, but la Cour et al conducted an experiment in porcine eyes that revealed that microcirculation of the retina is maintained regardless of a 10 to 20 mmHg decrease in MOPP [13]. Additionally, Kyhn et al, in a study on porcine eyes, found that ischemic damage of the retina occurred by decreased ocular perfusion pressure, and concluded that maintaining MOPP at 5 mmHg for 2 h can irreversibly damage retinal tissue [14].

In the present study, the mean difference between pre-injection IOP and IOP immediately following the injection was 27.01 ± 9.88 mmHg. The mean difference between pre-injection MOPP and MOPP just after injection was 21.25 ± 9.30 mmHg. The mean change in IOP and MOPP 30 min after the injection compared to the baseline IOP and MOPP were only 0.92 ± 3.44 mmHg and -0.53 ± 4.94 mmHg, respectively. Therefore, recovery of IOP and MOPP to the pre-injection values occurred within 30 min after the injection. Furthermore, no less than 10 mmHg of MOPP level was observed at different time-points (Table 3). These results indicate that intravitreal anti-VEGF injection is a safe treatment modality because the blood supply to the retina and optic nerve head was not impaired by the injection.

We assumed that the degree of change in post-injection IOP and MOPP would be affected by the difference in the injection dosage (0.05 mL Avastin, 0.07 mL Lucentis) and by the presence of post-injection vitreous regurgitation. These variables, however, had minimal effects on the difference in post-injection IOP and MOPP (Tables 4, 5). This outcome is thought to be the result of the relatively small sample size (65 eyes), the small difference in the injection dosage (0.02 mL), and a low incidence of post-injection vitreous regurgitation (11 eyes) due to the use of a 30-gauge needle.

In designing this study, it was predicted that the stressful situation of intravitreal injection would increase the MBP, resulting in an attenuation of the decrease in MOPP. Evaluation of serial changes in MBP revealed that pre-injection mean BP was 95.39 ± 9.16 mmHg and mean MBP immediately after the injection, and at 30 min, 1 day, and 1 week after the injection was 104.28 ± 8.92 mmHg, 95.93 ± 9.39 mmHg, 93.57 ± 8.54 mmHg, 94.27 ± 7.48 mmHg, respectively. Compared to the pre-injection MBP, MBP immediately after the injection increased by 9 mmHg, equivalent to the 6 mmHg increase in MOPP. Consequently, the rise in MBP right after the intravitreal Avastin or Lucentis injection can be considered to have a protective effect against the decrease in post-injection MOPP.

A previous report by Rasier et al. revealed an increase in blood pressure, which was checked 1 week, 3 weeks, and 6 weeks after intravitreal Avastin injection [15]. Rasier et al. also warned of the possible occurrence of systemic hypertension, which can be considered a systemic complication following intravitreal Avastin injection [15]. In the present study, however, there was no prominent increase in MBP during the 1 week following intravitreal Avastin or Lucentis injection except for the transient spike in MBP immediately after the injection. Additionally, the type of injected drug (Avastin versus Lucentis) did not have a statistically remarkable influence on the difference in MBP (*p* > 0.05).

## Conclusion

In conclusion, Most eyes in our study achieved normalization of IOP and MOPP within 30 min without need for any immediate intervention, such as paracentesis. Additionally, the decrease in MOPP caused by the increase in IOP did not induce an extremely low pressure level (< 10 mmHg). Based on these findings, the instant increase in IOP after intravitreal anti-VEGF injection does not cause a significant decrease in the blood flow of the retina and optic nerve head. Therefore, except for patients with glaucoma, uveitis, which can cause impaired ability of IOP regulation, repeated or prolonged IOP monitoring for normalization of IOP after intravitreal anti-VEGF injections is not necessary.

## Abbreviations

VEGF, vascular endothothelial growth factor; IOP, intraocular pressure; MOPP, mean ocular perfusion pressure; MAP, mean arterial pressure; MBP, mean blood pressure; DME, Diabetic macular edema; PDR, proliferative diabetic retinopathy; AMD, age-related macular degeneration; RVO, retinal vein occlusion; CSC, central serous chorioretinopathy; CNV, choroidal neovascularization.
